# T_FH_ cells accumulate in mucosal tissues of humanized-DRAG mice and are highly permissive to HIV-1

**DOI:** 10.1038/srep10443

**Published:** 2015-06-02

**Authors:** Atef Allam, Sai Majji, Kristina Peachman, Linda Jagodzinski, Jiae Kim, Silvia Ratto-Kim, Wathsala Wijayalath, Melanie Merbah, Jerome H. Kim, Nelson L. Michael, Carl R. Alving, Sofia Casares, Mangala Rao

**Affiliations:** 1 US Military HIV Research Program, Walter Reed Army Institute of Research; 2 Henry M. Jackson Foundation for the Advancement of Military Medicine, Bethesda, Maryland, USA; 3 US Military Malaria Vaccine Program, Naval Medical Research Center, Silver Spring, Maryland, USA

## Abstract

CD4^+^ T follicular helper cells (T_FH_) in germinal centers are required for maturation of B-cells. While the role of T_FH_-cells has been studied in blood and lymph nodes of HIV-1 infected individuals, its role in the mucosal tissues has not been investigated. We show that the gut and female reproductive tract (FRT) of humanized DRAG mice have a high level of human lymphocytes and a high frequency of T_FH_ (CXCR5^+^PD-1^++^) and precursor-T_FH_ (CXCR5^+^PD-1^+^) cells. The majority of T_FH_-cells expressed CCR5 and CXCR3 and are the most permissive to HIV-1 infection. A single low-dose intravaginal HIV-1 challenge of humanized DRAG mice results in 100% infectivity with accumulation of T_FH_-cells mainly in the Peyer’s patches and FRT. The novel finding of T_FH_-cells in the FRT may contribute to the high susceptibility of DRAG mice to HIV-1 infection. This mouse model thus provides new opportunities to study T_FH_-cells and to evaluate HIV-1 vaccines.

Follicular helper T cells (T_FH_) are a functionally distinct CD4^+^ T helper cell subset that play a major role in the induction of protective immunity against foreign pathogens. T_FH_ cells reside within the follicles of secondary lymphoid tissue and are characterized by the expression of CXCR5, ICOS, and PD-1 as well as the transcription factor B cell lymphoma-6 (BCL-6)^1^,^2^. In the germinal centers (GC), T_FH_ cells undergo a tight interaction with B cells and provide important signals for the induction and affinity maturation of antibody responses through the ligation with co-receptors such as ICOS, SLAM, and CD40L as well as cytokines including the signature T_FH_ cell cytokine IL-21^1^,^2^,^3^. Moreover, T_FH_ cells have been shown to be critically involved in immunoglobulin class switch recombination and maturation of B cell responses into memory B cells or long-lived plasma cells^4^,^5^,^6^,^7^,^8^. Previous studies have demonstrated that T_FH_ cells are susceptible to HIV and SIV infection, expand during chronic infection, and can serve as a reservoir for latent HIV infection^9^,^10^. Despite the predominant location of T_FH_ cells within lymphoid follicles, many studies of human T_FH_ cells have characterized cells in the peripheral blood^3^,^10^,^11^,^12^,^13^,^14^. Therefore, understanding the function and regulation of T_FH_ cells within lymphoid tissues, and the interaction between T_FH_ and B cells during chronic HIV infection, could be helpful in improving vaccine development strategies.

The mucosal tissues in the gut and FRT are permissive to HIV-1 infection and play a crucial role in HIV-1 transmission^15^,^16^,^17^. Similar to the gut associated lymphoid tissue (GALT)^16^, the genital mucosa has been shown to contain organized mucosa-associated lymphoid tissue (MALT) and large lymphoid aggregates^18^,^19^,^20^. However, it is currently unknown what role T_FH_ cells play in the mucosal tissue during HIV-1 infection. To study T_FH_ cells in the mucosal tissue before and after HIV-1 infection, we utilized a newly generated strain of humanized mice. These *NOD.Rag1KO.IL2RγcKO* mice express *HLA-DR0401* molecules (DRAG mice)^21^. DRAG mice are infused with HLA-DR matched human hematopoietic stem cells and unlike the BLT mice do not require human fetal liver and thymus transplants to generate human immune cells^21^,^22^.

In this study, we find a high level of reconstitution of human T and B cells in the gut, FRT, and spleen (SP) of humanized DRAG mice. T_FH_ cells are abundant in mucosal tissues of the gut [Peyer’s patches (PP), intraepithelial lymphocytes (IEL), and lamina propria lymphocytes (LPL)], and FRT of humanized DRAG mice. We find that CXCR3^+^ T_FH_ cells express the highest levels of IL-21 and IFN-γ. Furthermore, we find a strong correlation between the expression of CXCR3, PD-1, CCR5, and the permissiveness to HIV-1 infection. A single low dose intravaginal challenge with primary HIV-1 results in 100% infection rate in humanized DRAG mice with accumulation of T_FH_ cells mainly in the PP and FRT. The abundance of human effector CD4 memory T cells and the high accumulation of T_FH_ cells in the mucosal tissues of humanized DRAG mice makes this a suitable model to study HIV pathogenesis, the functional role of T_FH_ cells, and to evaluate candidate vaccines.

## Results

### DRAG m**i**ce are highly reconstituted with human CD45^+^ cells

**Figure 1:**
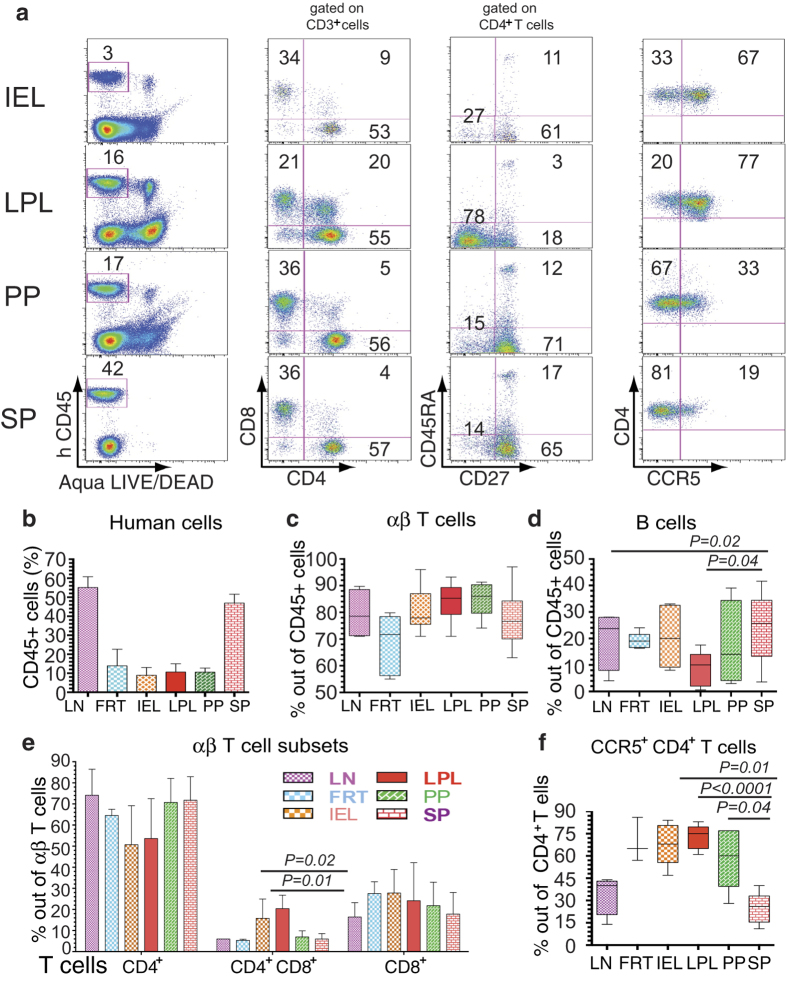
Characterization of human T cell reconstitution in the gut and lymphoid tissues of DRAG mice (**a**) Human hematopoietic cells from IEL, LPL, and PP were harvested from gut and from the SP of individual humanized DRAG mice (n = 3-8). A representative dot plot is shown. Live cells were selected based on Aqua LIVE/DEAD dye and on human CD45 to quantify human-cell reconstitution in each population (% of human CD45^+^ cells is shown in the left panel). CD3^+^ T cells were gated on human CD45^+^ cells and CD3^+^ T cells were stained for human CD4^+^ and CD8^+^ T cells (second panel). CD4^+^ T cells were stained for CD45RA and CD27 to determine the percentages of naïve CD4^+^ T cells (CD45RA^+^CD27^+^), effector memory CD45RA^**−**^CD27^**−**^), or central memory CD4^+^ T cells (CD45RA^**−**^CD27^+^) (third panel). The expression of CCR5 receptor on CD4^+^ T cells is shown in the last panel. (**b**) The reconstitution of human hematopoietic cells in the LN (n = 6), FRT (n = 5), gut (IEL, LPL, PP; n = 8)), and SP (n = 8) of humanized DRAG mice are shown as the average % of human CD45^+^ cells ± SEM. (**c**) and (**d**) show the distribution of human T cells and B cells, respectively in LN (n = 4), FRT (n = 5), IEL, LPL, PP (n = 5), and SP (n = 5). (**e**) The average ± SEM of single and double positive T cells are shown. (**f**) The percentage of CCR5^+^ CD4^+^ T cells in the secondary lymphoid tissues and the gut are shown (LN: n = 4; FRT: n = 3; IEL, LPL, PP: n = 5; SP: n = 5). In (**c**), (**d**), and (**f**) the horizontal bars in the box-and-whisker plots indicate the median values and the vertical lines indicate values from the lowest to the highest. The p values shown in d and f were calculated using Student’s t test.

To assess the level of reconstitution of human cells in DRAG mice, we harvested the gut (PP, IEL, LPL), FRT, LN, and SP. The presence of PP in DRAG mice, in contrast to other humanized mice, allowed us to characterize the lymphocytes in this tissue. Human cells were identified by the expression of human hematopoietic cell marker CD45 (Fig. 1a left panel, representative dot plot). All lymphoid and mucosal tissues investigated were reconstituted with human cells (Fig. 1a left panel, Fig. 1b, average percentage with standard error of mean from 5-8 separate experiments, and Supplementary Fig. 1a, representative dot plot). Furthermore, the reconstitution of human cells in the gut of DRAG mice was high, compared to other humanized mouse strains^23^. The frequency of T cells was 60–85% of the human CD45^+^ cells (Fig. 1c), while B cells constituted 10-35% of human CD45^+^cells (Fig. 1d and Supplementary Fig. 2). There were no significant differences in the frequency of CD4^+^ T cells among the various tissues investigated (Fig. 1e). Among T cells, the percentage of CD4^+^ T cells was much higher than CD8^+^ T cells (Fig. 1a second panel and Fig. 1e), which is similar to what has been observed in humans. A subpopulation of T cells that expresses both CD4 and CD8 coreceptors (CD4^+^CD8^+^ or double positive) were mainly present in LPL (about 20%) and IEL (15.8%), and also in the PP (7%), SP (6%), FRT (5.3%), and LN (6%), albeit at a lower frequency (Fig. 1a second panel, Fig. 1e, and Supplementary Fig. 1a). The frequency of CD4^+^CD8^+^ T cells in LPL and IEL was significantly higher compared to SP and LN (*P* = 0.01 and *P* = 0.02, respectively).

To identify the human CD4^+^ T cell subsets, cells were stained for memory and naïve markers (CD27 and CD45RA). The majority of CD4^+^ T cells (79–96%) exhibited a memory phenotype, which is defined as negative for the naïve CD4^+^ T cell marker CD45RA (Fig. 1a third panel). The majority of memory CD4^+^ T cells in LPL (Fig. 1a third panel) and FRT (Supplementary Fig. 1a) were negative for CD27 and CD45RA thus exhibiting an effector memory phenotype. In contrast, the majority of memory CD4^+^ T cells in IEL, PP, and SP (Fig. 1a third panel) displayed a central memory phenotype by expressing CD27 but not CD45RA. Since the coreceptor CCR5 is required for R5 HIV-1 strains^24^,^25^, we examined its expression on CD4^+^ T cells. The majority of CD4^+^ T cells in FRT, IEL, LPL, and PP expressed CCR5 (50–80%) compared to SP (25%), and LN (40%) (Fig. 1a fourth panel, Fig. 1f, and Supplementary Fig. 1a). The CCR5^+^CD4^+^ T cells were significantly higher (*P* < 0.04) in IEL, LPL, and PP compared to SP and LN.

**Figure 2:**
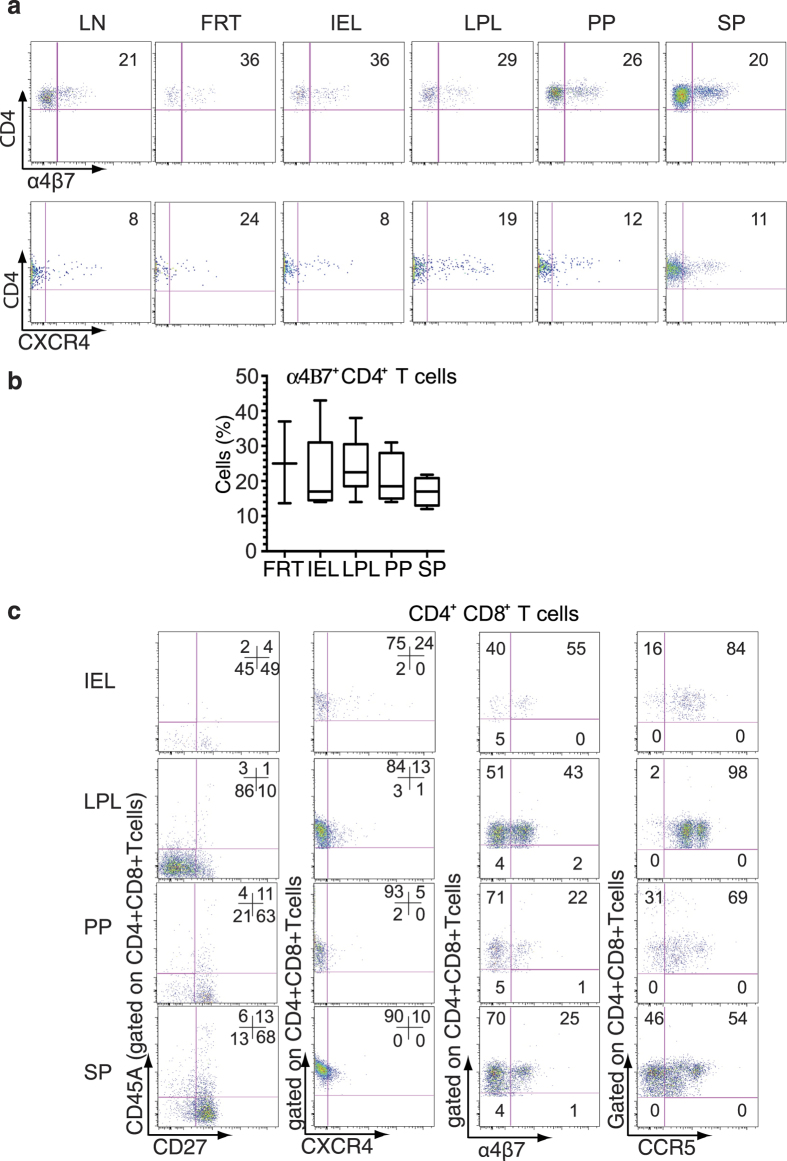
Tissue specific CD4^+^ and CD4^+^CD8^+^ T cells have distinct phenotypes (**a**) A representative dot plot shows the expression of α4β7 and CXCR4 receptors on CD4^+^ T cells (n = 2-5 separate experiments). Dead Cells were excluded by Aqua LIVE/DEAD dye, gated for human CD45 (hCD45), and then gated for CD3 followed by CD8 and CD4 T cells. The gated CD4^+^T cells were then analyzed for α4β7 and CXCR4 expression. (**b**) The frequency of α4β7^+^ CD4^+^ T cells from different tissues is shown. The horizontal bars in the box-and-whisker plots indicate the median values and the vertical lines indicate values from the lowest to the highest. (LN: n = 3; FRT: n = 3; IEL, LPL, PP: n = 5; SP: n = 4). (**c**) A representative dot plot of CD4^+^CD8^+^ T cell phenotype in IEL, LPL, PP, and SP is shown. The left panel depicts the memory phenotype based on the staining of gated CD4^+^CD8^+^ T cells for CD27 and CD45RA. The gated CD4^+^CD8^+^ T cells were also analyzed for CXCR4 (second panel), α4β7 (third panel), and CCR5 (fourth panel) expression, respectively. The experiment was done at least three times.

The expression of α4β7-integrin receptor is associated with trafficking to mucosal tissues through an interaction with its ligand mucosal addressin cell adhesion molecule-1^26^. α4β7 binds HIV-1 and facilitates HIV-1 infection of mucosal CD4^+^ T cells^27^,^28^. Our results show that mucosal tissues, which are the primary sites of HIV-1 transmission had CD4^+^α4β7^+^ T cells in varying frequencies (Fig. 2a upper panel and Fig. 2b). Similarly, X4-tropic HIV-1 strains use CXCR4 as a coreceptor^29^,^30^, and we found CXCR4 expression on FRT and LPL CD4^+^ T cells was higher compared to CD4^+^ T cells from other tissues examined (Fig. 2a lower panel).

Since CD4^+^CD8^+^ T cells (double positive) constitute a significant population of humanized DRAG mice gut tissue T cell subsets, we analyzed the memory phenotype and the expression of HIV-1 coreceptors CCR5, CXCR4, and α4β7 on these cells. CD4^+^CD8^+^ T cells in the tissues examined (Fig. 2c left panel) exhibited a memory phenotype similar to that of (single-positive) CD4^+^ T cells obtained from the same tissue (Fig. 1a third panel). The frequency of CXCR4^+^CD4^+^CD8^+^ T cells in IEL (Fig. 2c second panel) was higher compared to CXCR4^+^CD4^+^ T cells in IEL (Fig. 2a lower panel). Similarly, the proportion of α4β7^+^ CD4^+^CD8^+^ T cells in IEL and LPL (Fig. 2c third panel) was higher than α4β7^+^ CD4^+^ T cells (Fig. 2a upper panel). However, no significant differences were observed between the two populations in PP and SP. In contrast, the frequency of CCR5^+^CD4^+^CD8^+^ T cells (Fig. 2c fourth panel) is significantly higher (paired Student’s t test) than the frequency of CCR5^+^CD4^+^ T cells (Fig. 1a fourth panel) in the IEL *(P* = 0.04) and LPL (*P* = 0.02).

### T_FH_ cell subsets are enriched in the gut tissues

**Figure 3:**
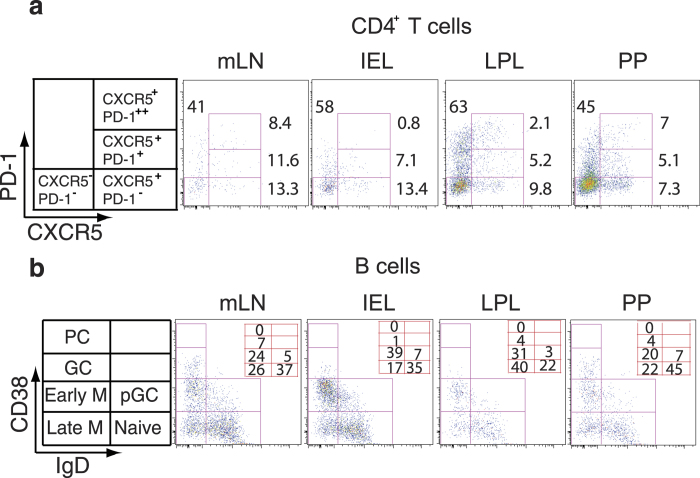
Distribution of T_FH_ cells and B cell subsets in mucosal tissues and lymphoid organs (**a**) Representative flow cytometry dot plots of CXCR5 and PD-1 expression on memory CD4^+^ T cells showing the distribution of CXCR5^+^PD-1^++^, CXCR5^+^PD-1^+^, and CXCR5^+^PD-1^−^ CD4^+^ T cells. Cells were gated for hCD45 and Aqua LIVE/DEAD then gated for CD3 followed by gating on CD8^+^ and CD4^+^ T cells. CD4^+^ T cells were further gated on CD45RA negative cells. The gated CD4^+^ CD45RA^**−**^ T cells were then analyzed for PD-1 and CXCR5 expression. The frequency of CD4^+^ T cell subsets is shown. (**b**) Distribution of B cell subsets. B cells (hCD45^+^CD19^+^ cells) were stained for IgD and CD38. Naive (CD38^**−**^ IgD^+^), pre-germinal center B cells (CD38^+^ IgD^+^), germinal center (CD38^++^ IgD^**−**^), early memory (CD38^+^ IgD^**−**^), late memory B cells (CD38^**−**^ IgD^**−**^), and plasma cells (CD38^+++^ IgD^**−**^) are shown in the various tissues analyzed. The numbers represent the frequency of each subset. PC = plasma cells, M = memory B cells, pGC = pregerminal, and GC = germinal center B cells. The experiment was done 3 times for both (**a**) and (**b**).

T_FH_ cells have been studied in the peripheral blood, tonsil, and lymph nodes^9^,^10^,^11^,^12^,^13^,^14^,^31^,^32^. However, the distribution of T_FH_ cells in the gut and FRT has not been investigated. Analysis of single cell suspensions from mesenteric lymph nodes (mLN), IEL, LPL, and PP of humanized DRAG mice revealed CXCR5^+^PD-1^++^ CD4^+^ T_FH_ cells (Fig. 3a). T_FH_ cells were largely present in the mLN (8.4%), and PP (7%), and at lower frequencies in IEL (0.8%), LPL (2.1%) (Fig. 3a), and FRT (3%) (Supplementary Fig. 3). We also found a small population of CXCR5^+^CD4^+^ T cells that were intermediate for PD-1 expression (CXCR5^+^PD-1^+^ CD4^+^ T cells). Mesenteric LN were included since they are enriched for GC and were used as a positive control in this experiment.

### Human B cell subsets in lymphoid tissue, gut, and FRT

A substantial advantage of humanized DRAG mice is the reconstitution of functional human B cells with the ability to perform class switch recombination and elicit antigen-specific IgG antibodies upon vaccination^21^. As this model may be suitable for testing future vaccines, we investigated the distribution of B cell subsets in various tissues. In our study, we found that the largest population of B cells had a naive phenotype (CD38^**−**^ IgD^+^) in all tissues examined except in LPL (Fig. 3b). A substantial population of B cells in all of the tissues was in the early (CD38^**+**^IgD^**−**^) and late (CD38^**−**^IgD^**−**^) memory phase, while a small subset of B cells had a GC phenotype (CD38^**++**^IgD^**−**^) (Fig. 3b).

### CXCR3^+^ T_FH_ cells predominantly produce IL-21

**Figure 4:**
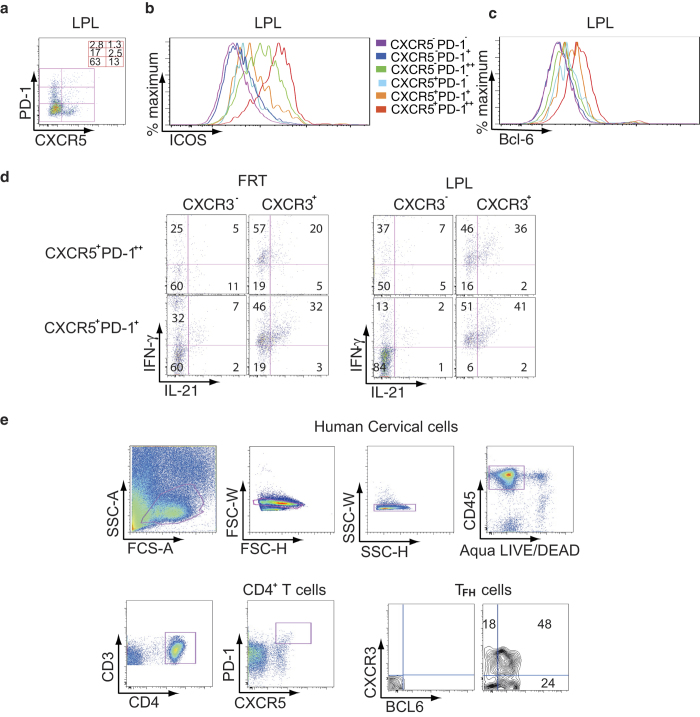
CXCR5^+^PD-1^++^ T_FH_ cells in the gut and FRT of humanized DRAG mice express ICOS and BCL-6 and produce IL-21 (**a**) A representative dot plot from two independent experiments of LPL depicts CD4^+^ T cells divided into 6 subsets based on CXCR5 and PD-1 expression. The frequency of CD4^+^ T subsets is shown. (**b**) and (**c**) Flow cytometry histograms from a representative experiment (n = 2) from LPL show ICOS (**b**) and BCL-6 expression (**c**) on CD4^+^ T cells. Cells were gated for hCD45 and Aqua LIVE/DEAD. Cells were then gated for CD3 and CD4. CD4^+^ T cells were gated for PD-1 and CXCR5 then for ICOS (**b**) and BCL-6 (**c**). (**d**) A representative dot plot from two independent experiments shows the cytokine production by T_FH_ cells from FRT and LPL. Cells were stained for hCD45, Aqua LIVE/DEAD, CD3, CD19, CD8, IgD, CD38, and then sorted for CD4^+^ T cells and B cells. In a second sort, CD4^+^ T cells were stained for PD-1 and CXCR5 to sort for CXCR5^+^PD-1^+^ and CXCR5^+^PD-1^++^ CD4^+^ T cells. B cells were sorted for memory cells expressing CD38^+^IgD^**−**^ cells. CXCR5^+^PD-1^+^ and CXCR5^+^PD-1^++^ CD4^+^ T cells were cultured with autologous memory B cells obtained from the same tissue. A schematic diagram of the sorting is shown in Supplementary Fig. 3. Stimulated FRT and LPL CD4^+^ T cells were stained for intracellular IL-21 and IFN-γ. (**e**) Endo- and ectocervix of human FRT were extracted from the muscular tissues and were digested with collagenase. The gating strategy is shown. Cells were gated for hCD45 and for Agua LIVE/DEAD followed by CD3 and CD4. CD4^+^ T cells were gated for PD-1 and CXCR5. CXCR5^+^PD-1^++^ T_FH_ cells were unstained (left panel) or stained for CXCR3 and BCL-6 (right panel). A representative dot plot from two independent human cervical tissues is shown.

It has been reported that a peripheral subset of CXCR5^+^PD-1^+^ CD4^+^ T cells, which are negative for CXCR3 correlate with neutralizing antibody levels in HIV-1 infected individuals^13^. Based on PD-1 and CXCR5 expression patterns, CD4^+^ T cells can be divided into CXCR5^**−**^PD-1^**−**^, CXCR5^**−**^PD-1^+^, CXCR5^**−**^PD-1^++^, CXCR5^+^PD-1^**−**^, CXCR5^+^PD-1^+^, and CXCR5^+^PD-1^++^ cells. The data for LPL are shown in Fig. 4a. Previous studies in humans have found that T_FH_ cells can also express ICOS during activation^33^,^34^, therefore we next analyzed for the expression of ICOS (Fig. 4b). While the majority of CXCR5^+^PD-1^++^ (red histogram) and CXCR5^**−**^PD-1^++^ (green histogram) CD4^+^ T cells expressed ICOS, a small proportion of CXCR5^+^PD-1^+^ cells (orange histogram) also displayed this receptor on their surface (Fig. 4b). BCL-6 is a transcription factor required for the differentiation of T_FH_ cells^4^. We found that CXCR5^+^PD-1^++^ CD4^+^ T cells also expressed high levels of BCL-6 with CXCR5^+^PD-1^+^ CD4^+^ T cells expressing intermediate levels of BCL-6 (Fig. 4c). These results indicate that the CXCR5^+^PD-1^++^ CD4^+^ T cells are T_FH_ cells while the CXCR5^+^PD-1^+^ CD4^+^ T cells are likely to be precursor T_FH_ (pre- T_FH_) cells.

The signature cytokine of T_FH_ cells is IL-21, which is critical for both B cell maturation and generation of memory response^1^,^35^. To investigate the ability of T_FH_ cells to activate memory B cells, we sorted CXCR5^+^PD-1^+^ and CXCR5^+^PD-1^++^ CD4^+^ T cells from FRT and LPL of humanized DRAG mice. In parallel, we sorted autologous memory B cells from the same tissues (Supplementary Fig. 3) and incubated them with sorted CXCR5^+^PD-1^++^ or CXCR5^+^PD-1^+^ CD4^+^ T cells. After stimulation with PMA and ionomycin, cells were stained for CXCR3 and for intracellular IL-21 and IFN-γ (Fig. 4d). Our data show that in both FRT and LPL, IL-21 and IFN-γ were mainly expressed by CXCR3^+^ CD4^+^ T cells, which were either CXCR5^+^PD-1^++^ or CXCR5^+^PD-1^+^. However, the proportion of intracellular IL-21^+^ cells was low in PD-1^++^ CXCR3^**−**^ and PD-1^+^CXCR3^**−**^ CD4^+^ T cells (Fig. 4d). The presence of T_FH_ cells in the FRT of humanized DRAG mice was unexpected, therefore, to confirm the presence of T_FH_ cells in human FRT, we obtained cervical tissue from routine hysterectomy and extracted the endocervix and ectocervix. The ecto- and endocervix were treated with collagenase to obtain the cellular fractions and then analyzed by flow cytometry. The endo- and ectocervix contained approximately 1–3% CXCR5^+^ PD-1^++^CD4^+^ T cells (Fig. 4e). The majority of these cells (about 72%) expressed BCL-6 confirming the presence of human T_FH_ cells (Fig. 4e) in human FRT.

### LPL and FRT cells are highly permissive to HIV-1

**Figure 5:**
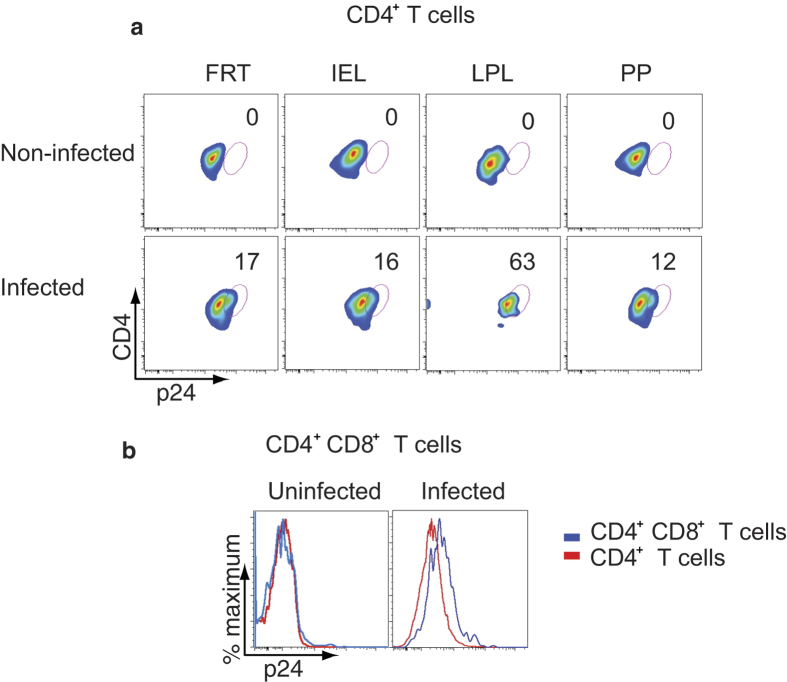
CD4^+^ T cells from FRT, IEL, LPL, and PP are highly permissive to HIV-1 infection. Stimulated cells from the indicated tissues were infected with HIV-1 (primary US-1) for 2 days. (**a**) Flow cytometry shows intracellular staining of p24 in CD4^+^ T cells of the indicated tissues (upper panel: uninfected cells; lower panel: HIV-1 infected cells). (**b**) A representative overlay histogram shows the intracellular p24 staining in HIV-1 infected CD4^+^CD8^+^ T cells (blue line) compared to CD4^+^ T cells (red line) obtained from LPL (left panel: uninfected cells; right panel: HIV-1 infected cells). Cells were stained for both intracellular and extracellular CD4. A representative plot of two independent experiments is shown in (**a**) and (**b**).

To determine if the reconstituted human cells in DRAG mice are susceptible to *in vitro* HIV-1 infection, cells were isolated from the FRT and gut (IEL, LPL, and PP), stimulated, infected with 2.2 × 10^3^ infectious units of HIV-1 (US-1, 0.5 ng of p24), and then cultured for 48 hours as described in the Materials and Methods Section. Cells were stained for CD3, intracellular p24, for intracellular and extracellular CD4, and then analyzed by flow cytometry (Fig. 5a). Cells from the gut mucosal tissues and FRT were infected with HIV-1 to varying degrees. CD4^+^ T cells from LPL were the most permissive (63%) to infection (Fig. 5a). The proportion of CD4^+^CD8^+^ T cells from LPL that were infected with HIV-1 was higher than CD4^+^ T cells (geometric mean fluorescent intensity = 689 vs. 530 respectively) obtained from the same tissue (Fig. 5b). The productivity of HIV-1 infection was confirmed by analyzing the culture supernatants for p24. Approximately 94–250 ng ml^−1^ of p24 was detected in the culture supernatants of HIV-1 infected FRT and gut cells (data not shown).

### CXCR3^+^ CCR5^+^ T_FH_ cells are highly susceptible to HIV-1

**Figure 6:**
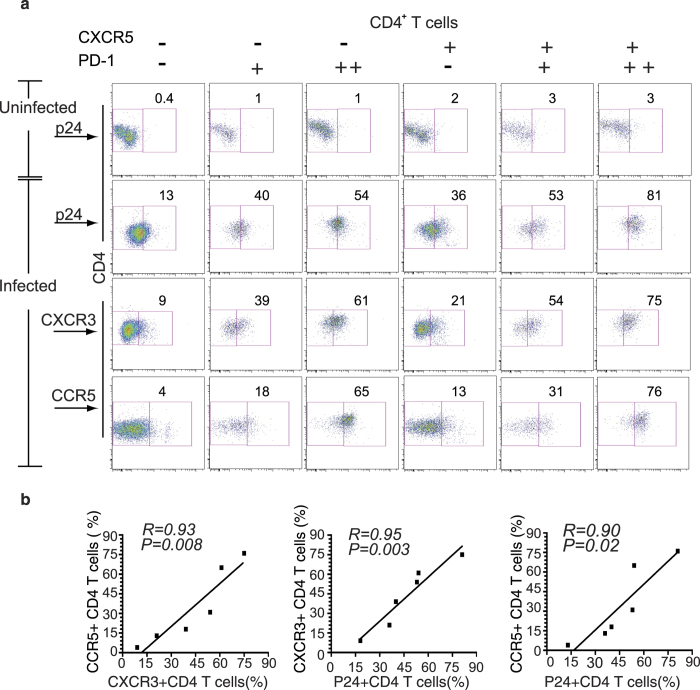
CXCR5^+^PD-1^++^ T_FH_ cells are highly permissive to HIV-1 infection. (**a**) LPL cells were stimulated with PHA for two days then left uninfected or infected with HIV-1 (primary US-1) for 3 days. HIV-1 infected CD4^+^ T cells were detected by intracellular staining with anti-p24 antibody. A representative flow cytometry dot plot showing p24 staining of non-infected CD4^+^ T cell subsets (top row); p24 staining of infected CD4^+^ T cell subsets (second row); CXCR3 positive CD4^+^ T cell subsets (third row) and CCR5 positive CD4^+^ T cell subsets (bottom row) are shown. Cells were stained for both intracellular and extracellular CD4. (**b**) Graphs show the correlation between the expression of CXCR3 and CCR5 (left), CXCR3 and intracellular p24 (middle) and CCR5 and intracellular p24 (right) CD4^**+**^ T cells from LPL. Linear regression, coefficient of correlation and P values are shown. Representative data from two independent experiments are shown.

To test the *in vitro* HIV-1 infectivity of CD4^+^ T cell subsets in IEL, LPL, and PP, cells were stimulated, either uninfected or infected with HIV-1, and then cultured. After culture, cells were stained and analyzed as mentioned above. Uninfected LPL are shown in Fig. 6a first row. CXCR5^+^PD-1^++^ CD4^+^ T cells were the most susceptible to HIV-1 infection (81%) compared to other subsets (Fig. 6a, second row, far right panel). Other CD4^+^ T cell subsets, CXCR5^**−**^PD-1^+^, CXCR5^**−**^PD-1^++^, CXCR5^+^PD-1^**−**^, and CXCR5^+^PD-1^+^ cells were also infected albeit at a lower frequency (36–55%) (Fig. 6a, second row). The CXCR5^**−**^PD-1^**−**^ CD4^+^ T cells appeared to be more resistant to infection (Fig. 6a, second row, far left panel). The fraction of cells expressing CXCR3 was the largest among CXCR5^+^ PD-1^++^ CD4^+^ T cells (Fig. 6a, third row, far right panel), and intermediate among the other subsets, except for CXCR5^**−**^PD-1^**−**^ cells, which displayed low expression of this receptor on the surface (Fig. 6a third row far left panel). The expression of CCR5 was high in the gut and FRT CD4^+^ T cells (Fig. 1a and Supplementary Fig. 1). Among CD4^+^ T cell subsets, CXCR5^+^PD-1^++^ and CXCR5^**−**^PD-1^++^ cells predominantly expressed CCR5 (65-76%), while CXCR5^**−**^PD-1^+^, CXCR5^+^PD-1^**−**^ and CXCR5^+^PD-1^+^ cells were intermediate for CCR5 expression (Fig. 6a fourth row). CCR5 receptor was detectable on a small subset (4%) of CXCR5^**−**^PD-1^**−**^ CD4^+^ T cells. We also found a strong correlation between CCR5 and CXCR3 expression (Fig. 6b left panel; *P* = 0.008 *R* = 0.93). Similarly, there was a strong correlation between HIV-1 infectivity (p24), CXCR3 (Fig. 6b middle panel; *P* = 0.003, *R* = 0.95) and CCR5 expression (Fig. 6b right panel; *P* = 0.02, *R* = 0.90). These results suggest that HIV-1 infectivity is correlated with CCR5 and CXCR3 expression on T_FH_ cells. To confirm that HIV-1 infection for 3 days did not contribute to the upregulation of PD-1 on CD4^+^ T cells, we sorted LPL cells from uninfected HIV-1 humanized DRAG mice using a BD FACS Aria cell sorter (Supplementary Figure 4). The sorted hCD45^+^CD4^+^CXCR5-PD^−^1^−^ cells were then infected in duplicate with primary HIV-1 as described in the Methods Section, washed, and then cultured for three days. We found that 7% (Supplementary Figure 4B, upper panel) and 10% (Supplementary Figure 4B, lower panel) of the cells were infected and remained negative for PD-1 expression. Based on these data, we conclude that HIV-1 infection does not upregulate the expression of PD-1 in the first 3 days of infection.

### Pre- T_FH_ and T_FH_ cells accumulate in LPL and FRT

**Figure 7:**
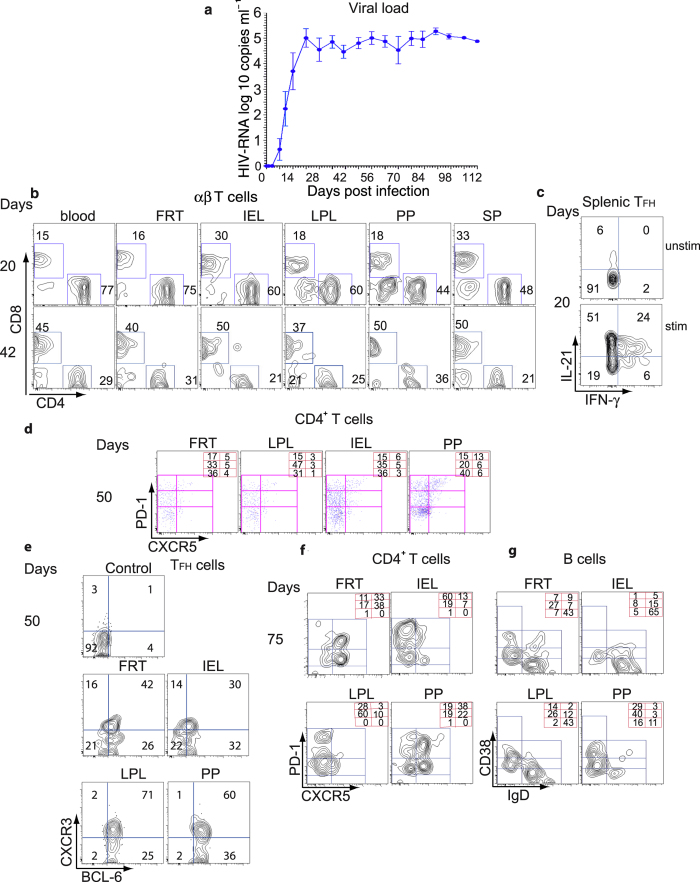
*In vivo* HIV-1 infectivity of DRAG mice and distribution of mucosal B and T cells after infection. (**a**) Viral load in the plasma of infected humanized DRAG mice. Humanized DRAG mice (n = 8) were intravaginally inoculated with a single dose of HIV-1 primary BaL (10,000 Infectious Units/mouse, 2.54 ng of p24). Individual plasma samples from DRAG mice before and after infection were analyzed for RNA viral load (copies per ml plasma) as mentioned in the Methods section using the Abbott RealTime HIV-1 Test. (**b**) A representative contour plot showing the depletion of human CD4^+^ T cells from the indicated tissues of HIV-infected humanized DRAG mice at 20 and 50 days post infection. (**c**) Frequency of IL-21 and IFN-γ producing CXCR5^+^PD-1^++^ T_FH_ cells from spleen of HIV-1 infected humanized DRAG mice at 20 days post infection. Cells were stimulated with PMA and ionomycin for 6 hours, permeabilized, and stained with anti–IL-21 and anti–IFN-γ antibodies. Upper panel; unstimulated cells (unstim.). Lower panel; stimulated cells (stim). (**d**) and **(e)** Cells were stained for PD1, CXCR5, BCL-6 (intracellular staining), and CXCR3. (**d**) A representative plot showing the distribution of T_FH_ and pre-T_FH_ cells in the indicated tissues 50 days post infection is shown. The frequency of CD4^+^ T cell subsets is shown in the right hand corner. (**e**) T_FH_ cells from (**d**) were then gated for BCL-6 and CXCR3 expression. Upper left panel shows T_FH_ splenic cells unstained for CXCR3 and BCL-6 (control). (**f**) A representative flow cytometry contour plot shows the distribution of T_FH_ CD4^+^ T cells. The numbers at the right hand corner refer to the frequency of CD4^+^ T cell subsets and (**g**) the frequency of B cell populations in FRT and gut tissues 75 days post infection. The representative flow cytometry contour plot of B cells is based on the expression of CD19, CD38, and IgD.

The distribution of human CD4^+^, CD8^+^ T cells, and B cells reconstituted in humanized DRAG mice is similar to that reported in humans, suggesting that the DRAG mice might be susceptible to HIV-1 infection. Mice (n = 8) were challenged intravaginally with a single low dose of purified primary HIV-1 BaL (2.54 ng p24 per mouse; 10,000 infectious units) and followed for 112 days (14 weeks). HIV-1 RNA was detected in the blood as early as 7 days after intravaginal challenge with the viral load steadily increasing up to day 21 (Fig. 7a). The viral load peaked on day 21 (5.3 × 10^5^ copies ml^−1^) and did not vary significantly thereafter (Fig. 7a). Mice were analyzed at various time points post infection to determine the ratio of CD4^+^/CD8^+^ T cells. When the mice were analyzed at 20 days post-infection (the first detectable copies of HIV-1 RNA were seen on day 13), the ratio of CD4^+^/CD8^+^ T cells was above 1 in all tissues (Fig. 7b, upper panels). When the mice were analyzed at 42 days post-infection, the ratio of CD4^+^/CD8^+^ T cells was below 1 (Fig. 7b, lower panels) and this ratio continued to decrease as the infection progressed. Mice euthanized at the end of the study, showed a large depletion not only in CD4^+^ T cells but also in CD8^+^ T and B cells in the mucosal tissues (data not shown). The decrease in CD8^+^ T and B cells has also been reported in the gut tissues of HIV-1 infected BLT mice^36^.

To determine if T_FH_ cells from the HIV-1 infected DRAG mice can also produce IL-21 and IFN-γ, splenocytes were stimulated with PMA and ionomycin and then analyzed by flow cytometry. Our results showed that splenic T_FH_ cells produced IL-21 and IFN-γ compared to unstimulated cells (Fig. 7c). We then determined the frequency of T_FH_ cells at various time points post infection (Fig. 7d and 7f). The frequency of T_FH_ cells varied among the tissues investigated (Fig. 7d). We found an increase in the frequency of T_FH_ cells in FRT from 5% to 33% and in PP from 13% to 38% on days 50 and 75 post infection, while the frequency did not change in LPL (Fig. 7d and 7f). CXCR5^+^PD-1^+^ pre-T_FH_ cells followed a similar pattern as was seen with the T_FH_ cells with an increase in the frequency in FRT from 5% to 38% and from 6% to 22% in PP (Fig. 7d and 7f). The percentage of CXCR5^+^PD1^++^CD4^+^ T cells expressing BCL-6 was highest in PP (96%) and LPL (96%) followed by FRT (68%) and IEL (62%) (Fig. 7e). Among this population, the frequency of T_FH_ cells that expressed CXCR3 varied from 30% to 71% (Fig. 7e).

B cell subsets were largely diminished in the gut tissues of humanized DRAG mice at 75 days post infection, (Fig. 7g). The highest frequency of naive B cells was seen in FRT, IEL, and LPL (43–65%), while it was lower in PP (11%). GC B cells were largely present in LPL (14%) and PP (29%) but at a reduced level in the FRT (7%), and rarely found in the IEL (1%) (Fig. 7g). A higher frequency of early memory B cells was found in PP (40%), FRT (27%), and LPL (26%). In contrast, the frequency of late memory B cells was greatly diminished in PP, LPL, IEL, and FRT.

## Discussion

In this study, we demonstrate that humanized DRAG mice have a high level of reconstitution of human B and T cells in the gut, FRT, and SP. This allowed us to study mucosal CD4^+^ T cells before and after HIV-1 infection in the gut and FRT with special emphasis on T_FH_ cells. CD4^+^ T cells from all three tissues expressed varying levels of HIV-1 coreceptors, CCR5, CXCR4, and α4β7. Mucosal tissues are the primary sites of HIV-1 transmission. The binding of the gut homing receptor α4β7 to gp120 potentially facilitates HIV-1 infection of mucosal CD4^+^ T cells^27^,^28^,^37^. Our study demonstrates a high frequency of α4β7^+^ and CCR5^+^ CD4^+^ T cell memory phenotype in LPL, IEL, and FRT, which likely increased their susceptibility to HIV-1. Furthermore, the expression of α4β7 and CCR5 was not restricted to CD4^+^ T cells but was also seen at much higher levels on CD4^+^CD8^+^ T cells, which has not been previously reported. The high expression of CCR5 and α4β7 on IEL and LPL CD4^+^CD8^+^ T cells compared to CD4^+^ T cells, suggests that the CD4^+^CD8^+^ T cells are more susceptible to HIV-1 infection. In fact, our study shows that acquisition of HIV-1 by CD4^+^CD8^+^ T cells was greater than CD4^+^ (single positive) T cells obtained from the same tissue. Since the distribution and the frequency of memory phenotype were similar in CD4^+^ T cells and CD4^+^CD8^+^ T cells, this indicates that in addition to their memory phenotype, the high expression of HIV-1 coreceptors also plays a crucial role in HIV-1 acquisition. It has been shown that CD4^+^CD8^+^ T cells are highly susceptible to SIV in the gut^38^ and HIV in the periphery^39^. It has been demonstrated that CD4^+^CD8^+^ T cells are higher in the gut of healthy individuals and animals than in lymphoid organs^40^,^41^,^42^,^43^,^44^,^45^,^46^ indicating that our findings in the gut of humanized DRAG mice are consistent with published data in humans. We are currently investigating the function of CD4^+^CD8^+^ T cells in relation to HIV-1 infection.

During the early stages of HIV-1 infection, T_FH_ cells are functional and provide adequate B cell help. However in the chronic phase, although T_FH_ cells have increased in number, their support for B cells is largely diminished due to exhaustion of CXCR5^+^PD-1^++^ T_FH_ cells^31^,^47^,^48^,^49^,^50^. The molecular basis for the exhaustion of T_FH_ cells has not been fully understood. It has been reported that CXCR5^+^PD-1^++^ T_FH_ cells have a high content of HIV-1 DNA and express the cell division marker Ki-67^10^. Therefore, the increase in the number of CXCR5^+^PD-1^++^ T_FH_ cells is probably due to the activation caused by HIV-1 infection, which leads to their proliferation. It has also been shown that blocking PD-1-PDL interaction *in vitro* partially restored anti-HIV-1 antibody production^31^,^51^. Similarly, in the SIV model, CXCR5^+^PD-1^++^ T_FH_ cell exhaustion was related to excessive signaling of PD-1, while disruption of PD-1-PDL interaction increased SIV-specific antibody responses^52^,^53^.

Our study shows that CXCR5^+^PD-1^++^ CCR5^+^ T_FH_ cells were the most permissive CD4^+^ T cell subset to HIV-1 infection and correlated with the expression of cell surface markers PD-1, CCR5, and CXCR3. We also found another population of CD4^+^ T cells that express CXCR5, intermediate levels of PD-1, low levels of ICOS and BCL-6, which are likely to be pre-T_FH_. Although, the role of T_FH_ cells in the LN and in the blood of HIV-1 infected individuals has been explored^10^,^12^,^14^,^31^, their contribution to HIV-1 infection in the gut mucosal tissues has not been investigated despite the fact that the gut and FRT are the initial sites of HIV entry and early pathogenesis. While the human endocervix and uterus have been shown to contain organized mucosa-associated lymphoid tissue (MALT) and large lymphoid aggregates, which are similar to gut associated lymphoid tissue (GALT)^18^,^19^,^20^, the presence of T_FH_ cells in FRT has not been previously described. In our study, we identified T_FH_ cells and pre-T_FH_ cells in the FRT and gut of humanized DRAG mice. After HIV-1 infection, the frequency of T_FH_ cells in FRT and PP increased. We also confirmed the presence of T_FH_ cells in human cervical tissue. The identification of T_FH_ cells in the FRT may therefore contribute to the early seeding of HIV-1 infection.

CXCR3 is largely expressed on mucosal T cells, has a crucial role in T cell infiltration to mucosal tissues during infection, and is increased on viral specific effector and memory T cells^54^,^55^,^56^,^57^,^58^,^59^. However, the role of CXCR3 expression on T_FH_ cells still not fully understood. Previous studies have reported that CXCR3^+^ CD4^+^ T cells are susceptible to HIV-1 infection and latency^60^,^61^,^62^. Our data show that the frequency of CXCR3^+^ cells was highest among the mucosal CXCR5^+^PD-1^++^ T_FH_ cells compared to the other CD4^+^ T cell fractions in the gut of humanized DRAG mice. These cells were also the most permissive to HIV-1 infection and are the main source of IL-21 after *in vitro* stimulation in contrast to CXCR3^**−**^ T_FH_ cells. Our data are consistent with the findings of Bentebibel *et al*, who demonstrated that CXCR3^+^ peripheral T_FH_ cells were the main source of IL-21^11^. In contrast, two other studies have shown either CXCR3^**−**^ peripheral T_FH_ cells or both CXCR3^**−**^ peripheral T_FH_ cells and CXCR3^+^ peripheral T_FH_ cells as the main source of IL-21^13^,^32^. Our results clearly demonstrate that the CXCR3^+^CXCR5^+^PD1^++^CD4^+^ T cells express BCL-6 and are thus T_FH_ cells.

The identification of CXCR5^+^PD-1^++^ T_FH_ cells in the gut and FRT of humanized DRAG mice prompted us to evaluate this mouse as an *in vivo* model for HIV-1 infection. Our data show that DRAG mice are highly susceptible to intravaginal infection with HIV-1. A single low dose intravaginal inoculation with primary HIV-1 (2.54 ng p24) was sufficient to infect 100% of humanized DRAG mice. Therefore the high susceptibility of humanized DRAG mice to HIV-1 infection after intravaginal inoculation is likely related to (i) the high reconstitution of human cells in the female reproductive tract; (ii) the dominance of CD4^+^ T cell memory phenotype in gut tissues and FRT, (iii) the high levels of expression of HIV-1 coreceptors, and (iv) the accumulation of T_FH_ cells in the gut and FRT. Thus, the humanized DRAG mice represent a suitable model to investigate HIV-1 pathogenesis and opens new opportunities to use this mouse model to study T_FH_ cells in the gut and FRT and to evaluate the efficacy of potential candidate HIV-1 vaccines.

## Methods

### Mouse strain

Humanized DRAG mice *[Rag1KO.IL2RγcKO.NOD (“NRG”) strain]* with chimeric transgenes encoding for HLA-DR*0401 [HLA-DRA/HLA-DRB1*0401]) fused to the *I-Ed MHC- II* molecule were generated as previously described^21^. Four to six week-old DRAG mice were infused with HLA-DR*0401-positive human stem cells^21^. Human cell reconstitution was periodically assessed in the peripheral blood samples. All animal procedures in this study were conducted under IACUC protocols (ID numbers: D07-10, 13-RET-34) approved by WRAIR/NMRC and in compliance with the animal Welfare Act and in accordance with the principles set forth in the “Guide for the Care and Use of Laboratory Animals,” Institute of Laboratory Animals resources, National Research council, National Academy Press, 1996. Human cord blood samples were obtained from the New York Blood Center.

### Antibodies and reagents

Prior to flow cytometry, cells from IEL, LPL, PP, SP, LN, mLN, and FRT were stained with the appropriate anti-human antibodies. The following antibodies and reagents were purchased from BD: BV450 and PE-Cy™7 anti- CD45 (clone HI30, 1:100), Alexa Fluor® 647 anti-IL-21 (3A3-N2.1, 1:50), BV711 and Alexa Fluor® 647 anti-CD279 (PD-1; clone EH12.1, 1:10), PerCp Cy5.5 and BV421- anti-CXCR5 (clone RF8B2, 1:10), APC-H7 anti-CD3 (clone SK7, 1:50), ECD and PerCP-Cy5.5 anti-CD8 (clone RPA-T8, 1:25), PerCP-Cy^TM^5.5 anti-IgD (clone IA6-2, 1:15), Alexa Fluor® 488 and PE-Cy™7 anti- Bcl-6 (clone K112-91, 1:50), and PE anti-CD45RA (clone HI100, 1:100), Alexa flour 700 anti-CD27 (M-T271, 1:50), PE anti-CD19 (clone SJ25-C1, 1:50), APC-conjugated anti-CD38 (clone HIT2, 1:25), PE anti-CD184 (CXCR4; clone 12G5, 1:10), Percp5.5 and Alexa Fluor® 488 anti- CD183 (CXCR3; clone IC6, 1:100), FITC anti-CD20 (clone 2H7, 1:50), BV605 anti- CD10 (clone HI10a, 1:20), APC anti-CD69 (FN50, 1:50), and brefeldin A. Alexa Fluor® 700 CCR5 (HEK/1/85a, 1:20), and V450 and BV711 anti-IFN-γ (4S.B3, 1:25), PerCP-Cy5.5 anti-CD278 (ICOS; clone D10.G4.1, 1:25) were from Biolegend. Qdot 605 anti-CD4 (clone S3.5, 1:350) was obtained from Invitrogen. PE anti-p24 (KC57-RDI, 1:20) was purchased from Beckman Coulter. Phorbol 12-Myristate 13-Acetate (PMA), ionomycin, monensin, phytohemagglutinin (PHA), and Staphylococcal Enterotoxin B, Collagenase VIII and DNAse Type I were purchased from Sigma-Aldrich. Aqua LIVE/DEAD stain was purchased from Invitrogen and recombinant human IL-7 was from PeproTech.

### Labeling of anti-α4β7 antibody (Act-1)

Act-1 antibody (anti-α4β7 antibody) was obtained through the AIDS Research and Reference Reagent Program, Division of AIDS, NIAID, NIH: α4β7 monoclonal antibody (cat #11718) from Dr. A. A. Ansari. The Act-1 antibody was labeled using the Lightning-Link Allophycocyanin-XL conjugation Kit (Innova Biosciences) according to the manufacturer’s instructions.

### Isolation of Lymphocyte from gut, FRT, and SP

The spleen (SP), lymph nodes (LN), mesenteric lymph nodes (mLN), entire gut, and the entire female reproductive tract (FRT) were isolated from humanized DRAG mice. Single cell suspensions of SP and mLN were prepared. Peyer’s patches (PP) were obtained by locating white patches and plucking off with forceps. Fecal matter and mucus were removed from the mouse gut and FRT, tissues were cut into small fragments, and rinsed with cold PBS. Intraepithelial lymphocytes (IEL) were extracted by adding IEL extraction buffer (1x HBSS, 1x HEPES, 10% FBS, 5 mM EDTA) to gut tissue. Lamina propria lymphocytes (LPL) and FRT cells were obtained using digestion buffer (RPMI, 1x HEPES, 10% FBS, Collagenase VIII (100 du ml^−1^=100x) and DNAse Type I (0.1 mg ml^−1^). Cells from IEL, LPL, PP, SP, LN, mLN, and FRT from individual humanized DRAG mice were layered on top of a discontinuous percoll gradient (40% and 70%), and centrifuged at 850 g for 20 minutes at 4 °C. Cells were retrieved from the intermediate density fraction, washed in cold PBS, and processed for flow cytometry and/or for cell cultures. Cells were stained with fluorochrome-conjugated antibodies (6-11 color panel) and AQUA LIVE/DEAD stain, and were acquired on a 4-laser LSRII flow cytometer (BD Immunocytometry Systems). Electronic compensation was performed with antibody capture beads (BD Biosciences). Data were analyzed using FlowJo Version 9.7.6 (TreeStar).

### Analysis of T_FH_ cells in human cervical tissue

Per Walter Reed Army Institute of Research (WRAIR) Policy 12-09, submission to the WRAIR Institutional Review Board (IRB) or Human Subjects Protection Branch (HSPB) for review/approval was not required for this project, as de-identified (coded), pre-existing specimens from routine hysterectomy were purchased/obtained from a commercial source, The National Disease Research Interchange (Philadelphia, PA). Informed consent was obtained from all subjects. Briefly, mucosal epithelium and underlying stroma of both ecto- and endocervix were separated from muscular tissue^63^,^64^, dissected into tiny pieces, then treated with collagenase IV for 30 min and shaking at 37 °C. After digestion, cell suspension was loaded on a percoll gradient (35% to 70%) and the intermediate layer was taken, stained for T_FH_ cells and analyzed by flow cytometry.

### HIV-1 purification

HIV-1 stock of primary isolate BaL or US-1 (clade B) were grown in human peripheral blood mononuclear cells, purified, and stored as previously described^65^,^66^,^67^,^68^. The amount of p24 in the purified virus and the infectivity of the virus were determined using the HIV-1 p24 Antigen Capture Assay kit (ABL) and performing the P4R5 MAGI assay^69^.

### *In vitro* infectivity of cells

Freshly isolated percoll gradient purified cells were stimulated for 48 hours with PHA (5 μg ml^−1^) and recombinant human IL-7 (2 ng ml^−1^). We used IL-7 instead of IL-2 since IL-7 promotes and IL-2 inhibits the development of T_FH_ cells^13^,^70^,^71^. After 2 days of stimulation, cells were washed and infected with HIV-1 (US-1, clade B, 0.5 ng per well) for 1 hour or incubated with RPMI1640 media and then washed. The infected and non-infected cells were cultured in RPMI1640 media containing IL-7 (2 ng ml^−1^) for an additional 2-3 days and then analyzed by flow cytometry for surface and intracellular CD4 and intracellular p24. Supernatants harvested from HIV-1 infected cells were assayed in triplicate for the presence of p24 using HIV-1 p24 Antigen Capture Assay kit.

### B and T-cell co-culture and intracellular cytokine staining

LPL, IEL, and PP cells harvested after percoll gradient were stained with antibodies against hCD45, CD3, CD8, and CD19, labeled with Aqua LIVE/DEAD stain, and then sorted for B and CD4 T cells using a FACSAria. In a second consecutive sort, hCD45^+^CD45RA^**−**^CD3^+^CD8^**−**^CD19^−^ CD4 T cells were further stained with anti-PD-1 and CXCR5 to sort for CXCR5^+^PD-1^+^ and CXCR5^+^PD-1^++^ CD4^+^ T cells. Sorted hCD45^+^ CD3^−^CD8^−^CD19^+^ B cells were further stained with anti-IgD and anti-CD38 to sort for IgD^**−**^CD38^+^ memory B cells. Sorted CXCR5^+^PD-1^+^ or CXCR5^+^PD-1^++^ CD4^+^ T cells were cultured with sorted autologous memory B cells obtained from the same tissue. Cells (300-3000) from each population were cultured at a 1:1 ratio. Cells were stimulated with Staphylococcal Enterotoxin B (SEB, 100 ng ml^−1^) in 96-well U-bottom plates in complete RPMI 1640 medium containing 10% heat inactivated FBS as previously described^32^. Media was changed every 2 days. On day 7, cells were harvested, washed, and stimulated (10^6^ cells per ml) with 100 ng ml^−1^ PMA and 1 μg ml^−1^ionomycin for 6 hours and monensin (3 μM) was added to the cultures for the final 4 hours at 37 °C. To measure cytokine production by T_FH_ cells in the spleen of HIV-1 infected mice, cells (10^6^ per ml) were stimulated with 1 µg ml^−1^ PMA and 1 μg ml^−1^ ionomycin, and 100 ng ml^−1^ brefeldin A for 6 hours at 37 °C and 5%CO_2_. Cells were labeled with Aqua LIVE/DEAD stain kit for 30 minutes at RT followed by blocking the Fc receptors for 15 minutes at 4 °C. Cells were then stained at 4 °C for 30 minutes with appropriate antibodies, fixed, and permeabilized for 15 minutes each at RT with Cytofix/Cytoperm (BD), washed and stained at RT for 30 minutes with anti-IFN-γ and anti-IL-21. The frequency of cytokine-producing CD4 T cell populations was assessed by flow cytometry using a LSRII.

### Intravaginal inoculation of humanized DRAG mice with HIV-1

Female humanized DRAG mice were injected (2.5 mg per 50 μl per mouse) subcutaneously with medroxyprogesterone (Greenstone LLC) 7 days prior to infection. On the day of infection, mice were anesthetized and infected through the intravaginal route with HIV-1 BaL (10,000 Infectious Units 2.54 ng^−1^ of p24 per mouse) in a maximum volume of 20 μl. A sterile micropipette tip was used to deliver the virus and care was taken to ensure that no mucosal abrasions or tearing occurred.

### Assessment of viral load

Blood samples were collected from humanized DRAG mice pre- and post-infection every 3 days for two weeks and then at weekly intervals for a total of 16 weeks. Viral load in the whole blood was determined using the Abbott RealTime HIV-1 Test (Abbott Molecular, Inc.) with minor modifications. Whole blood specimens were collected in tubes containing EDTA solution and then frozen. To determine viral load, frozen blood samples were thawed and lysed. HIV-1 RNA was extracted following the FDA cleared test method and quantified. Although whole blood was used, no inhibition of the assay was observed based upon the performance of the internal extraction control. As the blood volumes collected were slightly variable, the values obtained were adjusted by 1 log to account for an average dilution factor of 10.

### Statistical analysis

Data are summarized as mean ± SEM or graphically displayed as box and whisker plots. Statistical analysis was performed using Prism Version 6.0c software (GraphPad). Data were analyzed using Student’s t test. Spearman’s rank test was used for correlation studies. *P* < 0.05 was considered statistically significant.

## Additional Information

**How to cite this article**: Allam, A. *et al.* T_FH_ cells accumulate in mucosal tissues of humanized-DRAG mice and are highly permissive to HIV-1. *Sci. Rep.* **5**, 10443; doi: 10.1038/srep10443 (2015).

## Supplementary Material

Supplementary Information
