# Supplementary material for: TFH cells accumulate in mucosal tissues of humanized-DRAG mice and are highly permissive to HIV-1
Source: Sci Rep. 2015 Jun 2;5:10443. doi: 10.1038/srep10443 (PMC4451806; doi:10.1038/srep10443)
Supplement: Supplementary Information [file srep10443-s1.pdf]

T_{FH} cells accumulate in mucosal tissues of humanized-DRAG mice and are highly permissive to HIV-1

Atef Allam[1,2*], Sai Majji[3], Kristina Peachman[1,2], Linda Jagodzinski[1], Jiae Kim[1,2], Silvia Ratto-Kim[1,2], Wathsala Wijayalath[3], Melanie Merbah[1,2], Jerome H. Kim[1], Nelson L. Michael[1], Carl R. Alving[1], Sofia Casares[3] and Mangala Rao[1*]

[1]US Military HIV Research Program, Walter Reed Army Institute of Research and [2]Henry M. Jackson Foundation for the Advancement of Military Medicine, Bethesda, Maryland, USA. [3]US Military Malaria Vaccine Program, Naval Medical Research Center, Silver Spring, Maryland, USA;

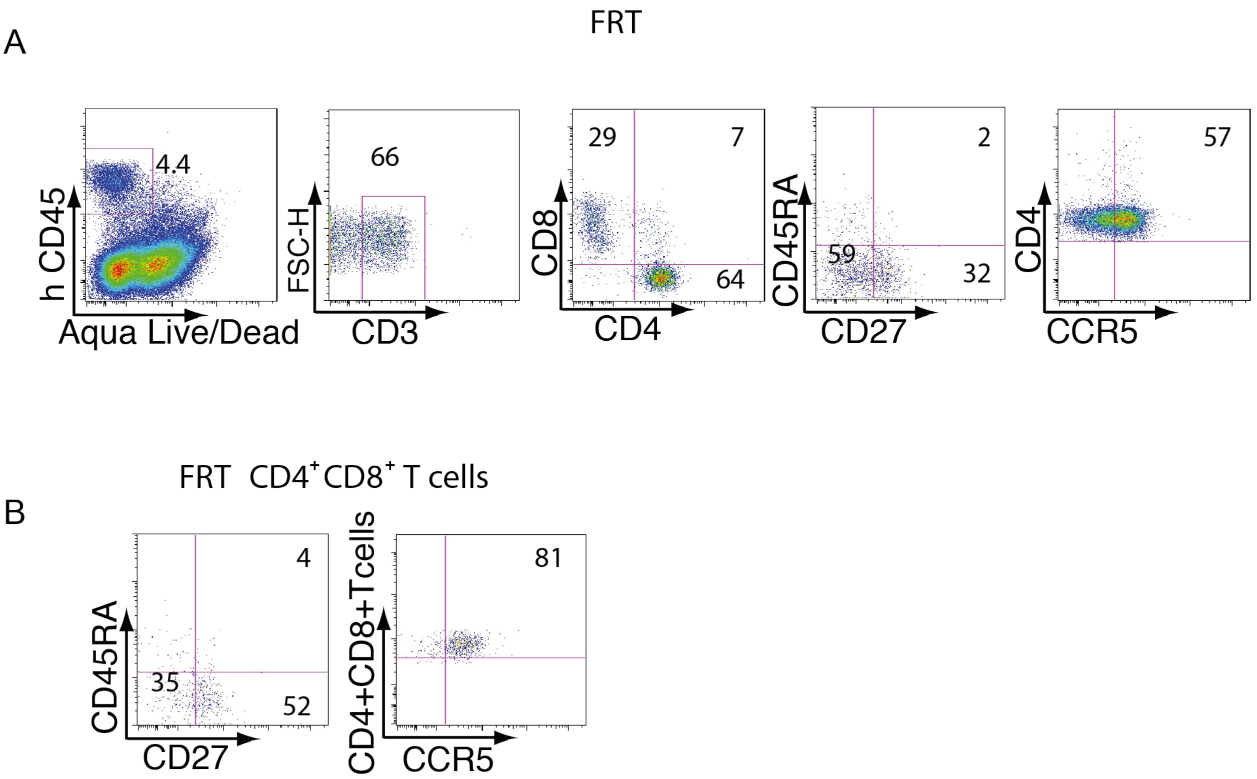

Supplementary Figure 1. The reconstitution of human cells in FRT of humanized DRAG mice. (**a**) A representative flow cytometry plot shows the reconstitution of human cells in the FRT of a DRAG mouse. Cells were stained with Aqua LIVE/DEAD and anti-hCD45 antibody (left panel). The frequency of T cells is shown in the second panel. Gated $CD3^+$ T cells were stained for CD4 and CD8 (third panel). $CD4^+$ T cell memory and naïve subsets were identified based on the expression of CD45RA and CD27 (fourth panel), and the frequency of CCR5 expressing CD4 T cells is shown in the right panel. (**b**) Shows the memory phenotype of $CD4^+CD8^+$ T cells (left panel). The gated $CD4^+CD8^+$ T cells were analyzed for CCR5 expression (right panel).

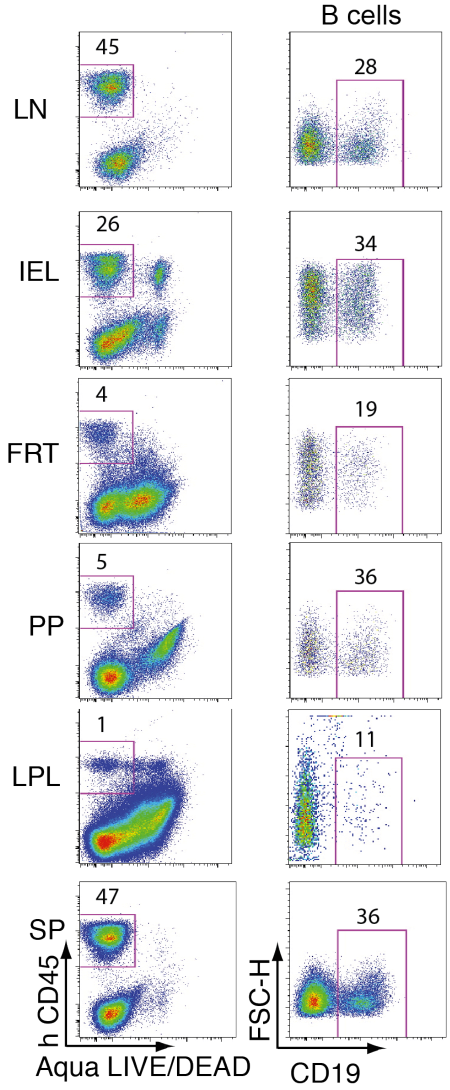

Supplementary Figure 2. Distribution of human B cells in the indicated tissues.

A representative flow cytometry plot depicts the proportion of human B cells in the indicated tissues of a humanized DRAG mouse. Cells were stained with Aqua LIVE/DEAD and anti-hCD45 antibody (left column), and were gated for CD19 expressing cells (B cells) (right column).

A

LPL
PP → Isolate single cell suspension → Load on Percoll gradient
FRT

Stain for human CD45 and CD19 → Sort-I: sort B cells (CD45+CD19+) and CD45+ CD19- cells

Stain B cells for CD38 and IgD → Sort-II: sort for memory B cells (CD38+IgD-)

Stain CD45+CD19- cells with CD45RA, CD3, CD8, PD-1 and CXCR5
→ Sort-III sort for CD45RA+CD3+CD8- CXCR5+PD-1++
or CD45RA+CD3+CD8- CXCR5+PD-1+ cells

B

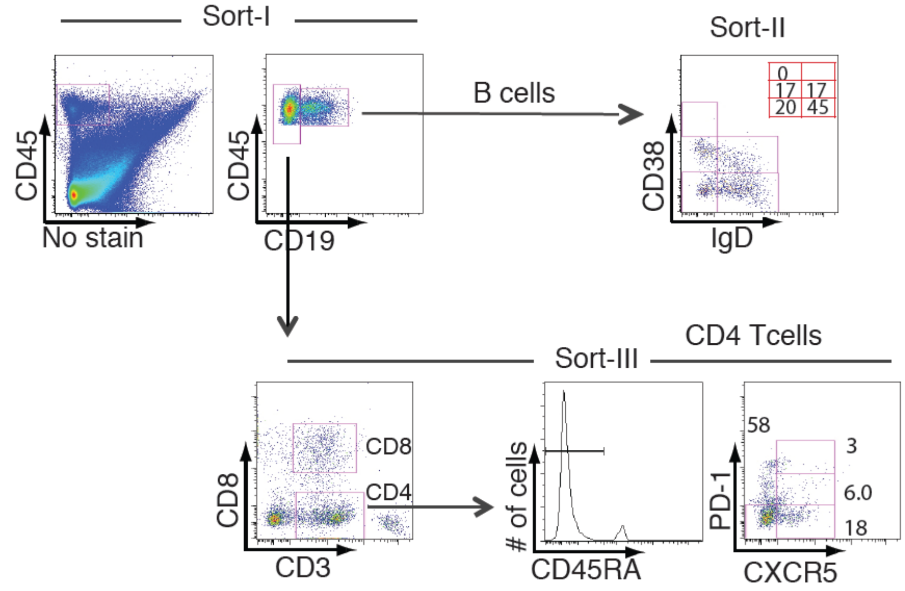

Supplementary Figure 3. Cell sorting procedure for FRT. (**a**) A schematic for the cell sorting procedure fro FRT and LPL is shown on the top. (**b**) A representative flow cytometry dot plot sort for B and T_{FH} cells is shown for FRT of a humanized DRAG mouse. The entire sorting experiment was performed twice with tissues pooled from 2 mice in each case.

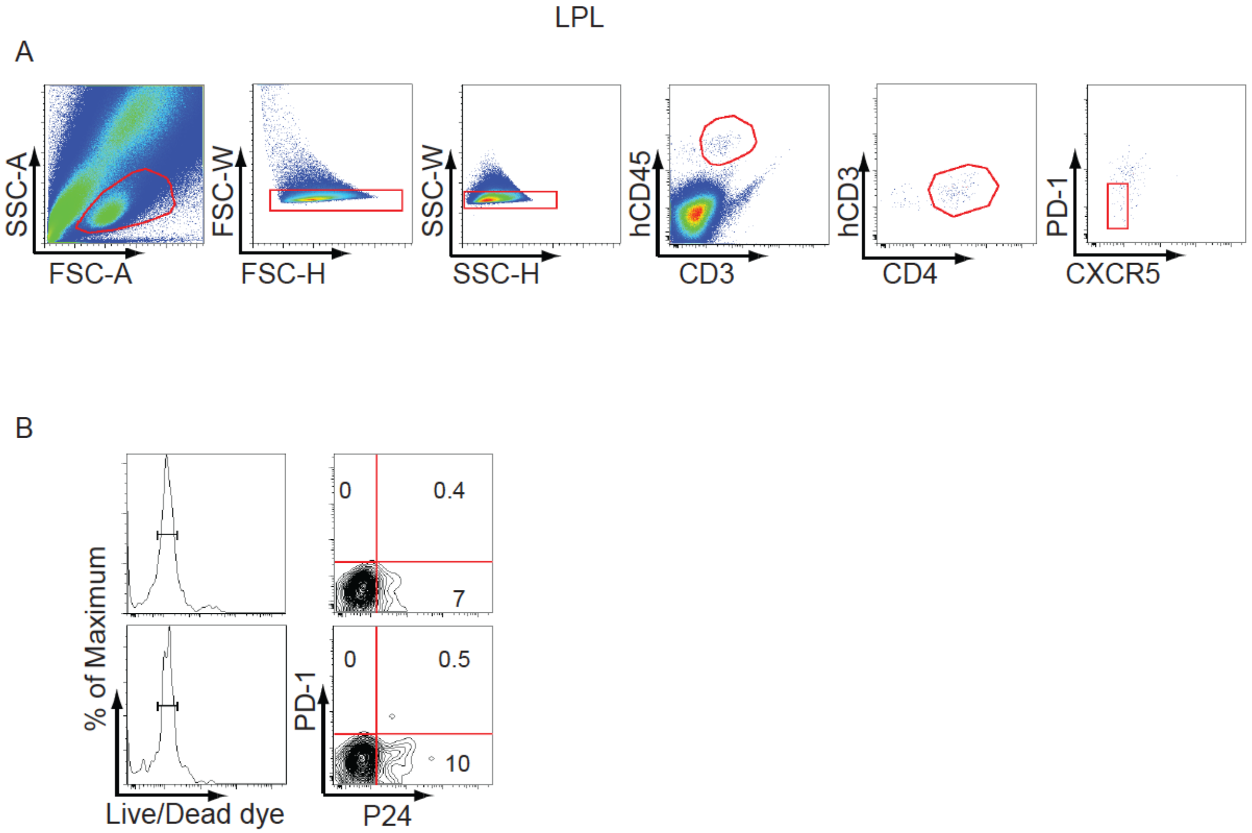

Supplementary Figure 4. HIV-1 infection does not upregulate PD-1 expression during early infection. (**a**) Cells were harvested and pooled from the LPL of 4 humanized DRAG mice then separated on a percoll gradient. Cells were stained for hCD45, CD3, CD4, PD-1 and CXCR5 and sorted using BD FACS Aria. Sorted cells (hCD45$^+$CD3$^+$CD4$^+$PD-1$^-$CXCR5$^-$) were washed then infected with primary HIV-1 for 3 days as indicated in the Methods section. (**b**) Cells in duplicate were labeled with Aqua Live/Dead dye, washed, fixed, permeabilized, stained for PD-1, CXCR5, and p24, and then analyzed on a LSRII. Cells were gated for Aqua Live/Dead dye and then live cells were gated for PD-1 and p24.
